# Sport-Related Human Activity Detection and Recognition Using a Smartwatch

**DOI:** 10.3390/s19225001

**Published:** 2019-11-16

**Authors:** Zhendong Zhuang, Yang Xue

**Affiliations:** The School of Electronic and Information Engineer, South China University of Technology, Guangzhou 510640, China; eezhuangzhendong@mail.scut.edu.cn

**Keywords:** sport-related activity monitoring, interval generation, periodic matching, convolutional neural network

## Abstract

As an active research field, sport-related activity monitoring plays an important role in people’s lives and health. This is often viewed as a human activity recognition task in which a fixed-length sliding window is used to segment long-term activity signals. However, activities with complex motion states and non-periodicity can be better monitored if the monitoring algorithm is able to accurately detect the duration of meaningful motion states. However, this ability is lacking in the sliding window approach. In this study, we focused on two types of activities for sport-related activity monitoring, which we regard as a human activity detection and recognition task. For non-periodic activities, we propose an interval-based detection and recognition method. The proposed approach can accurately determine the duration of each target motion state by generating candidate intervals. For weak periodic activities, we propose a classification-based periodic matching method that uses periodic matching to segment the motion sate. Experimental results show that the proposed methods performed better than the sliding window method.

## 1. Introduction

With the widespread use of wearable smart devices, sport-related activity monitoring using inertial sensors has become an active area of research, and it is used to improve the quality of life and promote personal health. In previous studies, the activity monitoring problem has often been defined as a human activity recognition (HAR) task [1]. An HAR model usually consists of two main parts—data segmentation and activity classification. The segmentation procedure uses a fixed-length sliding window to divide the sensor signal into different segments. The subsequent classifier is then used to classify these activities by using the information in these segments. Many researchers [2,3,4,5] have used this segmentation method to identify activities with strong periodicity and a single motion state because, after segmentation, each segment contains a meaningful motion state. However, many sports are characterized by complex motion states and non-periodicity. If such an activity signal is segmented with this sliding window, there is no guarantee that each segment will contain a useful motion state. Some segments may contain only noise. Therefore, for complex activities, an activity monitoring algorithm needs the ability to detect the duration of each meaningful motion state and then identify them.

**Figure 1:**
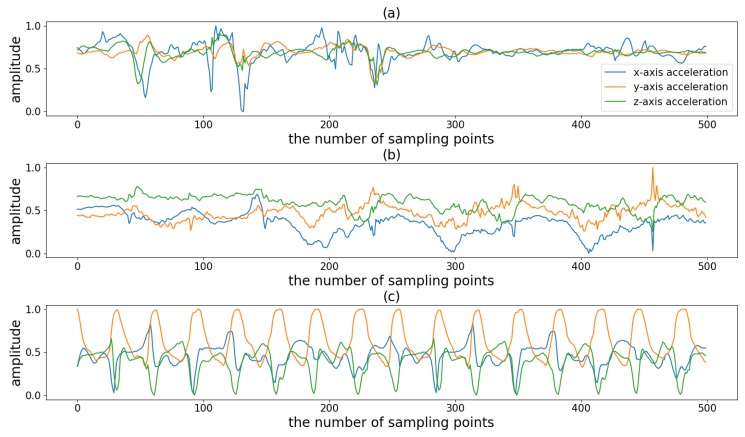
Illustration of triaxial acceleration of three kinds of sports: (**a**) the waveform of playing badminton, (**b**) the waveform of swimming, and (**c**) the waveform of running.

There are many kinds of sports and in this study we divided them into three types according to their periodicity and complexity. Figure 1 shows an example of three sports. The first type is the non-periodic activity with complex motion states (NP_CMS), such as badminton and basketball. When playing this type of sport, people do not repeat the periodic motion state but randomly switch between various motion states. For example, when playing badminton, people randomly switch between serving, swinging, smashing and other motion states. In other words, the activity involves multiple motion states, each of which does not loop but instead transitions to another state. The difficulty in monitoring this type of sport is accurately detecting the duration of different motion states. The second type is the activity with weak periodicity and complex motion states (WP_CMS). This type of activity also has multiple motion states and each state may occur in a continuous loop or may be converted to another state. Moreover, the cycle is not fixed and may vary from person to person. Even for the same person, the cycle may change at different times. For example, when swimming, people change their swimming strokes from time to time. Moreover, even if the same stroke is repeated, the frequency of the motion is not uniform. Because the motion state of these activities changes frequently, for better monitoring it is important to accurately determine the duration of each cycle for each motion state. The third type is the activity with strong periodicity and a single state, such as walking and jogging. This type of activity contains only one motion state, and the cycle of the motion state is more stable.

In previous HAR studies, most researchers have used the sliding window method to segment the motion signal and obtain good recognition performance. Ponce et al. [6] classified some daily activities, including sitting, standing, lying down, lying on one side and playing basketball. They used a 5 s sliding window to divide the sensor signal containing the target activity into different signal segments for training and test sets. They then trained an artificial hydrocarbon network to classify these labeled segments. Although their algorithms successfully identified basketball (a non-periodic activity with complex motion states), they only recognized that the user was playing basketball. We believe that meaningful motion states (such as shooting and smashing) should be accurately detected and identified to better monitor activities such as basketball. Attal et al. [7] applied four classification algorithms to classify 11 daily activities, such as standing, sitting, sitting down, and lying down. They also used a sliding window to segment the sensor signal and selected the segments containing the target activities for training and testing. Because they lacked training signal segments containing non-target activities, they were easily misclassified. Therefore, for these non-periodic activities, we propose a method that first detects the target motion states and then identifies them. This method can effectively detect the target motion state and reduce misclassification.

For activities with weak periodicity and complex motion states (such as swimming), many researchers have explored the sliding window-based activity recognition task. Siirtola et al. [8] applied an linear discriminant analysis (LDA) classifier to identify swimming strokes by acceleration data sampling at 5 Hz; then, they counted the number of each stroke using the peak count. Jensen et al. [9] compared the performance of four classification methods on the basis of different sliding window lengths and sampling rates. Brunner et al. [10] also used a sliding window to divide the sensor signal and then employed a convolutional neural network (CNN) as a classifier to recognize swimming strokes. They all had good recognition performance but they used a fixed-length sliding window to divide the swimming signal, which resulted in an inconsistent number of cycles per window and a different signal at the beginning of each window. These differences increase the difficulty of correctly classifying these movements. In particular, when the sliding window contains two strokes, the movement is easily misclassified. Therefore, in this work, we propose a classification-based periodic matching method to accurately detect one period of each stroke (motion state). First, a single-cycle candidate motion state signal is detected and inputted into a classifier. The classification result is then used to adjust the detection to obtain a more accurate period segmentation. In Section 4.2.1, the experimental results show that the recognition performance could be improved by combining the periodic matching method. Therefore, in this paper, we regard the monitoring of NP_CMS and WP_CMS activities as a human activity detection and recognition (HADR) task.

In recent years, the CNN [11] has developed rapidly and is widely used to improve the performance of HAR [12,13]. This indicates that CNNs can effectively extract the local dependency and interrelation between sensor signals. However, most algorithms simply use CNNs as a classifier and do not give them more deformation and expansion functions, as in the field of computer vision [14,15,16,17]. For example, in the field of object detection, Ren et al. [18] skillfully applied two convolutional layers instead of fully connected layers and proposed the region of interest (RoI) pooling layer to turn target detection tasks into the generation and recognition of candidate boxes. Inspired by this, we believe that the monitoring of the activity with non-periodic and complex motion states can be understood as the detection and recognition of target motion states in a long time-series. Using Faster R-CNN [18], we developed a candidate interval generation method in Reference [19]. In contrast to our previous work, in this study, we added adaptive components that allow our network to process sensor data of any length. By generating candidate intervals, our network can accurately determine the full duration of a single motion state for activity detection. Activity recognition is then implemented using these candidate intervals. The proposed approach is called the interval-based activity detection and recognition method. The experimental results show that this method reduces the difficulty and improves the accuracy of recognition. Furthermore, most of the work in activity detection and recognition can be achieved by reusing the same neural network, which greatly reduces the computational cost.

Additionally, we believe that the weak periodicity of the second type of activity can be utilized to detect the duration of each motion state. Therefore, we propose an adaptive detection method based on periodic matching. The method first finds a signal containing more than one period of motion state and divides it into several signals that contain only one period. A CNN is then used for identification and the periodic matching is adaptively adjusted by the classification result. We call this approach the classification-based periodic matching method.

We only found two public datasets containing non-periodic activity with complex motion states, namely, Physical Activity Monitoring for Aging People dataset (PAMAP2) [20] and Daily and Sports Activity dataset (DSA) [21]. Both of them lack annotations of the duration of meaningful motion states. Therefore, we collected two activity datasets (described in Section 2.1) for the two kinds of sports studied in this work. Instead of professional sports monitoring, we focus on providing convenient daily sports-related activity monitoring. Considering that smartwatches are widely used as wearable devices for remote health monitoring and are user-friendly [22,23,24], we chose smartwatches as activity acquisition devices.

In this paper, we focus on the detection and recognition of two complex activities. In summary, this research makes the following contributions. First, for the non-periodic activity with complex motion states, we propose a motion state detection and recognition method based on interval generation. Second, for the activity with weak periodicity and complex motion states, we propose a recognition method based on periodic matching. Finally, we conducted experiments on two datasets to demonstrate that the proposed methods can effectively detect the target motion states and improve recognition performance.

## 2. Datasets and Data Pre-Processing

### 2.1. Datasets

**Figure 2:**
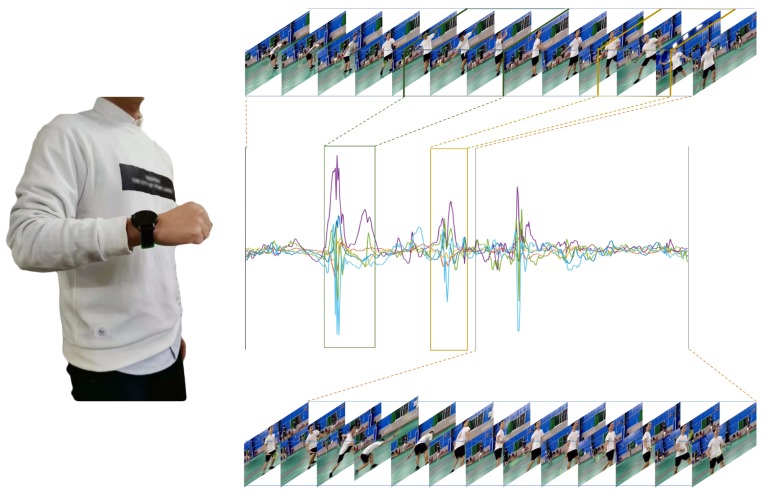
The graph shows the correct way to wear the watch, as well as the form of the data used in this paper. The left side shows the proper way to wear the watch. The middle of the right side shows the waveform of sensor signals collected by the watch during badminton. On the top and bottom of the waveforms are real-time pictures of the wearer’s movements. In particular, the waveform in the green box corresponds to the serving movement, the waveform in the yellow box corresponds to the swinging movement, and the frames corresponding to these two motions are framed by the corresponding dotted colored line.

For the training and verification of the model, we collected two datasets (the badminton dataset and the swimming dataset) for benchmarking. We used a smartwatch (Pacewear, as shown in the left part of Figure 2) to collect activity datasets. This watch can record triaxial acceleration and triaxial angular velocity (as shown in the middle of the right part of Figure 2), which are used by our algorithm for HADR. The sampling frequency of the Pacewear watch is 50 Hz, which is the sampling rate often used in HAR studies [1,5].

For the non-periodic activity with complex motion states (NP_CMS), we chose badminton as the target sport, with serving and swinging as target motion states. Specifically, 12 participants (8 men and 4 women) were asked to wear smartwatches to play badminton. For each participant, we recorded approximately 5 min of data accompanied by synchronized video recording. In the later stage, volunteers marked the specific start and end time-points of serving and swinging on the sensor data by referencing the video recording. Then, the signal was cut into units of 10 s and, finally, the signal fragments with the target motion states were selected as samples. Each signal fragment was annotated with the occurrence time of the target states and the order of participants.

For the activity with weak periodicity and complex motion states (WP_CMS), we chose swimming as the target sport, with freestyle, breaststroke, backstroke and butterfly stroke as the target motion states. Sixteen participants were asked to wear smartwatches while swimming back and forth three or four times for each stroke in a 25-m lane. Samples of each stroke were stored separately. Later, we labeled each set of samples with the motion state and the serial number of the participants.

### 2.2. Data Pre-Processing and Input Adaptation

As described in Section 2.1, the embedded sensor in the watch provides six-dimensional raw signals, including triaxial acceleration and triaxial angular velocity. All of them are used by our proposed detection and recognition methods. We normalized each one-dimensional signal separately. Then, a Gaussian filter was used to smooth the data and reduce noise.

The triaxial acceleration and triaxial angular velocity form a multi-dimensional time series. The data need to be converted into a “virtual image” for the CNN to process the time series—this process is called input adaptation. In general, each dimensional data is superimposed on a different channel to conduct one-dimensional convolution, or the data of different dimensions are tiled into a virtual 2D graph to adopt 2D convolution. In this work, we used the second approach.

Specifically, for the NP_CMS sport data, we added two lines of zero between the triaxial acceleration and the triaxial rotational angular velocity in a process called “zero padding” [25]. This effectively avoids the disturbance caused by different characteristics of the two kinds of signals during feature extraction.

## 3. The Proposed Method for Complex Activity Detection and Recognition

In this section, we propose different detection and recognition methods for two different activities. For the NP_CMS activity, we propose an interval-based detection and recognition method. For the WP_CMS activity, a periodic matching-based detection and recognition method is proposed.

### 3.1. NP_CMS Activity Monitoring

**Figure 3:**
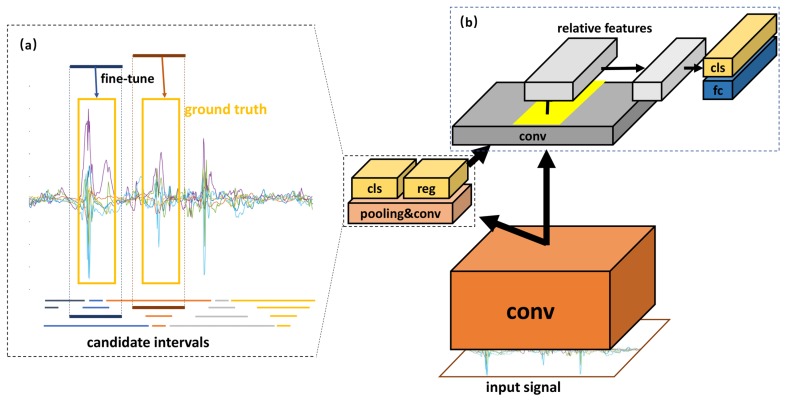
The flowchart of non-periodic activity with complex motion states (NP_CMS) activity detection and recognition. (**a**) Interval generation: the bottom lines with different colors and lengths represent the candidate anchors that we preset. The bold lines show the candidate interval. This step picks out the candidate interval from the anchors by using cls and fine-tunes it with reg to better match the ground truth. (**b**) Interval-based activity recognition: the yellow part of the gray feature map represents the relative features of the second candidate interval. The relative features are extracted from the feature map and then pooled into a fixed size. Finally, the fixed-size features are entered into the fully connected layer and softmax layer to produce the final recognition result.

Figure 3 shows the flowchart of the NP_CMS activity detection and recognition method. The method consists of two main parts—interval generation (activity detection) and interval-based activity recognition.

According to the translation variance of a CNN [26], Faster R-CNN finds the spatial correspondence between the input image and the feature map of the CNN output. It first presets many candidate boxes on the input image and then proposes a region proposal network (RPN) to use one feature map to score all candidate boxes at the same time. When the CNN input changes from images to time series, the correspondence is inherited by the time domain. Thus, we propose an interval generation-based human activity detection and recognition method. First, a number of candidate anchors are preset for the input signal, represented by lines of different colors and lengths, as shown in Figure 3a. These candidate anchors are responsible for predicting the duration of meaningful motion states. Then, the feature map of the CNN output is converted to cls and reg. The cls scores all the candidate anchors and selects the candidate intervals that match the ground truth. The reg fine-tunes the selected candidate intervals to generate results that are closer to the ground truth. Finally, the motion states in the fine-tuned candidate interval are classified, and the recognition result is obtained. It is worth noting that when performing the classification task, we extracted the relative features of the candidate intervals on the feature map according to the translation variance and used them for the classification, as shown in Figure 3b.

#### 3.1.1. Interval Generation

**Figure 4:**
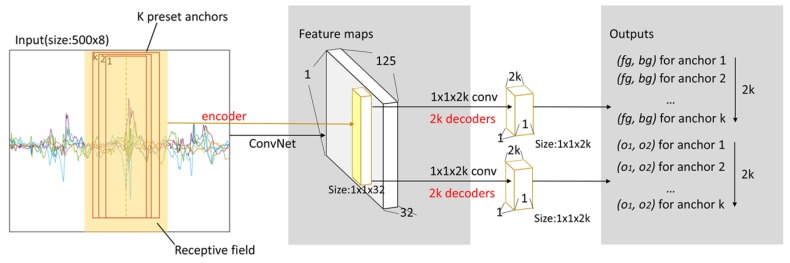
The illustration of interval generation.

To generate high-quality candidate intervals, we preset many interval anchors. Determining the approach that efficiently grades and fine-tunes the preset anchors is a difficult problem. According to the translation variance, we believe that the feature corresponding to each point on the feature map contains high-level semantic information corresponding to the relative receptive field of the input signal (the feature and its relative receptive field are marked in yellow in Figure 4). If the anchor is in the center of the receptive field, then the relative feature can be used to score and fine-tune it. Thus, we preset a set of anchors of different sizes in the center of the receptive field corresponding to each feature.

For the input sequence, the CNN is used to extract high-level semantic features and obtain the feature maps. Next, a 1 × 1 convolutional layer processes these feature maps to generate scores and offset parameters. Here, the CNN and 1 × 1 convolutional layer can be considered an encoder and a decoder, respectively. As shown in Figure 4, the CNN encodes the signal segment on the receptive field into 32-dimensional features on feature maps. Then, for *k* anchors on the receptive field, four 1 × 1 convolutional layers decode these features into 2k scores and 2k offset parameters. The generated scores and offset parameters are used to determine whether the anchors match the target motion states and fine-tune them further.

Each anchor has four parameters, which are denoted by $\left(fg,bg,o_{1},o_{2}\right)$. fg and bg are the probabilities that the anchor is foreground (IOU > 0.5) or background, while $o_{1}$ and $o_{2}$ are only valid for foreground boxes and are used to fine-tune them.

The candidate interval $\left(x_{sta}^{*},x_{end}^{*}\right)$ with offset parameters is calculated as follows:

(1) $$\begin{array}{cc} & x_{sta}^{*}=\left(1+o_{1}+0.5e^{o_{2}}\right)x_{sta}+\left(-o_{1}-0.5e^{o_{2}}\right)x_{end}\\ & x_{end}^{*}=\left(-o_{1}-0.5e^{o_{2}}\right)x_{sta}+\left(1+o_{1}+0.5e^{o_{2}}\right)x_{end} \end{array}$$

where $\left(x_{sta},x_{end}\right)$ represent boundary points of the foreground interval, and $\left(o_{1},o_{2}\right)$ represent the shift and scale.

**Anchor Design**

In this subsection, we detail the method of presetting the anchors.

1. As mentioned above, a set of anchors of different sizes are set in the center of the receptive field corresponding to each feature. For the alignment, the length of the input signal must be an integer multiple of the stride of the CNN output with respect to the input signal. On this basis, we set the input signal to a multiple of 4 (the stride of Net_1, which is used for interval generation) by adding zero to the end of it. Let $l_{os}$ be the length of the original signal; the number of zeros added (denoted by $N_{zs}$) can be obtained by the following calculation: $N_{zs}=\left\lceil \frac{l_{os}}{4}\right\rceil \times 4-l_{os}$. Therefore, our CNN can process the input of any length.
2. The adjusted signal is divided into *N* equal parts ($N=\frac{l_{a}}{4}$, where $l_{a}$ is the length of the adjusted signal). For each part of the signal, we set up a set of anchors with different lengths centered on the signal’s center. Therefore, we can get $\frac{l_{a}}{4}\times N_{a}$ anchors, where $N_{a}$ is the number of anchors in each set. In this work, we set the length of anchors to [16, 24, 32, 40, 48, 56, 64, 72, 80, 96], so these anchors can match each ground truth.

**Non-Maximum Suppression (NMS)**

This subsection addresses the reduction of redundant candidate intervals that match the ground truth but are not optimal. We adopted the non-maximum suppression (NMS) method to further screen the candidate intervals. The specific steps are as follows:

1. Sort all candidate intervals according to their scores and select the candidate interval with the highest score.
2. Calculate the IOU between the selected interval and each of the other candidate intervals. Remove the candidate intervals with an IOU that is larger than the threshold.
3. Repeat the above operations for the remaining candidate intervals until the last iteration, when the scores of all candidate intervals are less than the threshold.

In our algorithm, we set the threshold of NMS to 0.3.

#### 3.1.2. Interval-Based Activity Recognition

As mentioned above, for the input sequence, many candidate intervals are generated. However, it is inefficient to sample the signals in each candidate interval and then feed them into the CNN for classification. Thus, we improved the efficiency by sharing convolution. This process consists of three steps, as shown in Figure 3b. First, the features are extracted from the whole input sequence through the convolutional neural network. Then, features in the corresponding position of each candidate interval are cut from the convolved feature map according to the projection relation. Finally, the processed features are classified through the subsequent average pooling layer, fully connected layer, and softmax layer.

The key to this process is the approach to obtaining the corresponding position of each candidate interval in the convolved feature map. In contrast to other deep CNNs, our network is relatively shallow and spatially sensitive. Therefore, the projection relation between the input and the convolved feature map can be approximated by the case in which the feature map is obtained by sampling the signal at a certain sampling rate.

Because our network includes a fully connected layer, the input to the subsequent network must be the same size. For extracting regional features from the global feature map and then pooling them, we refer to the RoI pooling of Faster R-CNN. Let the length of the convolved feature map be $l_{f}$, the length of the original signal be $l_{os}$, and the coordinates of the candidate interval be $\left(x_{sta}^{*},x_{end}^{*}\right)$. Then,

(2) $$\begin{array}{cc} & x_{sta}^{f}=\langle \frac{x_{sta}^{*}\times l_{f}}{l_{os}}\rangle \\ & x_{end}^{f}=\langle \frac{x_{end}^{*}\times l_{f}}{l_{os}}\rangle \end{array}$$

where $\left(x_{sta}^{f},x_{end}^{f}\right)$ represents the position of the feature corresponding to the proposal in the feature map. After obtaining the features, they are subjected to adaptive pooling, i.e., average pooling with a variable step size. Since the input of the subsequent network is fixed at $l_{s}$, the step size is $\langle \frac{x_{end}^{f}-x_{sta}^{f}}{l_{s}}\rangle$.

#### 3.1.3. The Loss of CNNs

CNNs need to be trained by combining a loss function and a stochastic gradient descent method [27]. Our method for the detection and recognition of the first type of activity includes two convolutional neural networks, as described in Section 4.1. For Net_1, the loss consists of two parts, which are the anchor score and offset loss.

(3) $$L_{1}=L_{score}+L_{offset}$$

where $L_{score}$ is the softmax cross-entropy loss [28] over the background and foreground. When the *i*th anchor can match one or more real action intervals, its probability of being background and foreground is $y_{i}^{bg}=0$ and $y_{i}^{fg}=1$, respectively, and vice versa. Its function is described by the following equation.

(4) $$L_{score}=-\frac{1}{N_{an}}\sum_{i=1}^{N_{an}}y_{i}^{bg}\log\left(p_{i}^{bg}\right)+y_{i}^{fg}\log\left(p_{i}^{fg}\right)$$

where $p_{i}^{fg}$ and $p_{i}^{bg}$ respectively represent the probability of the *i*th sample of foreground and background estimated by CNN. $N_{an}$ is the number of total anchors.

As mentioned above, only offset parameters for candidate intervals are generated, so non-candidate intervals are ignored. This is achieved by multiplying the loss value of each anchor sample by $y_{i}^{fg}$. For an effective anchor $\left(x_{i,sta},x_{i,end}\right)$, the offset parameters $\alpha_{i}^{gt}$, $\beta_{i}^{gt}$ are first calculated with Equation (5), and then the loss values of the offset parameters $\alpha_{i}$ and $\beta_{i}$ are calculated with Smooth L1 loss [29].

(5) $$\begin{array}{cc} & \alpha_{i}^{gt}=\frac{x_{i,end}^{gt}+x_{i,sta}^{gt}-x_{i,end}-x_{i,sta}}{x_{i,end}+x_{i,sta}}\\ & \beta_{i}^{gt}=\log\left(\frac{x_{i,end}^{gt}-x_{i,sta}^{gt}}{x_{i,end}-x_{i,sta}}\right) \end{array}$$

(6) $$Smooth_{L_{1}}\left(x\right)=\left\{\begin{array}{cc} 0.5x^{2} & -1<x<1\\ |x|-0.5 & \mathrm{otherwise} \end{array}\right.$$

where $\left(x_{i,sta}^{gt},x_{i,end}^{gt}\right)$ are the start point and endpoint of the real interval, which has the highest value of IOU with the *i*th anchor. Thus, $L_{offset}$ is defined as follows:

(7) $$L_{offset}=\frac{\sum_{i=1}^{N_{an}}y_{i}^{fg}\left(Smooth_{L_{1}}\left(\alpha_{i}-\alpha_{i}^{gt}\right)+Smooth_{L_{1}}\left(\beta_{i}-\beta_{i}^{gt}\right)\right)}{\sum_{i=1}^{N_{an}}y_{i}^{fg}}$$

For Net_2, we used the cross-entropy loss. The label of each candidate interval is obtained from its best matching real interval (if it does not match any real interval, its label is “None”). We assume that we have *C* target actions, so the label of the *i*th anchor is $Y=\{y_{i,1},y_{i,2},...,y_{i,c},y_{i,c+1}\}$ ($y_{i,c+1}$ represents the class “None”). When the real label is the *c*th class, $y_{i,c}=1$. Otherwise, it equals 0. Then,

(8) $$L_{2}=-\frac{1}{N_{can}}\sum_{i=1}^{N_{can}}\sum_{k=1}^{c+1}y_{i,k}\log p_{i,k}$$

where $N_{can}$ is the number of total candidate intervals, and $p_{i,k}$ is the probability of *i*th candidate interval of the *c*th class, which is predicted by the CNN.

### 3.2. WP_CMS Activity Monitoring

**Figure 5:**
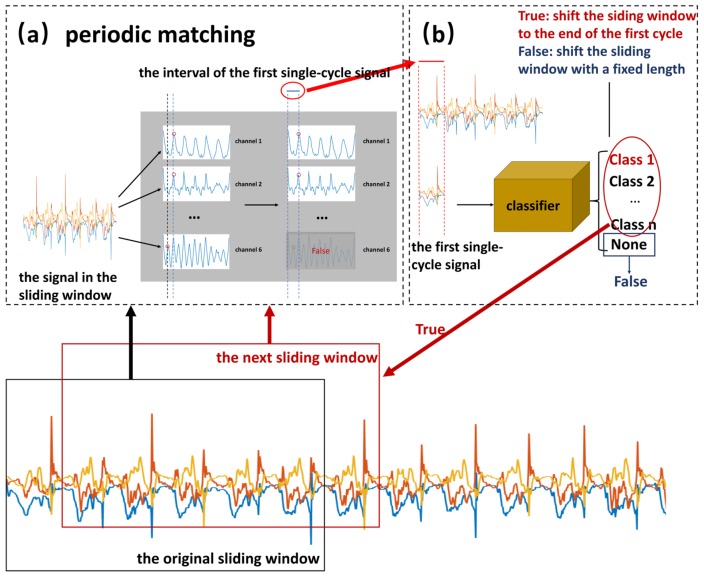
The flowchart of weak periodicity and complex motion states (WP_CMS) activity detection and recognition. (**a**) The pipeline of periodic matching method. (**b**) The pipeline of recognition method.

For the weakly periodic activity, although the movement period under the same or different motion states varies greatly (as shown in Figure 5), the period remains the same between several adjacent movements. From this characteristic, we propose a detection and recognition method based on periodic matching. The flowchart is shown in Figure 5. First, the sliding window obtains a fixed-length signal from the original input signal. Second, the first single-cycle signal from the input is detected by using periodic matching, as illustrated in Figure 5a. Third, a classifier is used to recognize the detected signal. If it is recognized as a certain motion state, the result of periodic matching is true; otherwise, it is false. Then, the different results are used to set different step sizes to get the next sliding windows as the new input, and steps 1–3 are repeated until the end of the original signal. Since this process requires the assistance of classification, it is called classification-based periodic matching. Finally, all the detected single-cycle signals are fed as input to a classifier to identify their motion states. In the experiment, five machine learning methods that have been successfully used in activity recognition were considered classifiers: CNN, k-nearest neighbor (KNN) [30], naive Bayes (NB) [31], random forest (RF) [32], and support vector machine (SVM) [33].

#### 3.2.1. Classification-Based Periodic Matching

The purpose of periodic matching is to determine the periodicity of the signal and extract the single-cycle signal. Autocorrelation refers to the cross-correlation between a signal and itself at different points in time. The autocorrelation sequence of the periodic signal is also periodic. On the basis of this characteristic, we propose a periodic matching algorithm to detect the single-period signals in a six-dimensional sensing signal $S=\{S_{1},S_{2},S_{3},S_{4},S_{5},S_{6}\}$. Periodic matching consists of two steps: calculating the autocorrelation and extracting the single-cycle signal on the basis of the classification.

**Calculating the autocorrelation**

A fixed-length *N* of a signal is extracted (it usually takes 2–3 times the maximum cycle of the target motion state). Thus, the autocorrelation $R^{j}=\left\{R_{1}^{j},R_{2}^{j},R_{3}^{j},R_{4}^{j},R_{5}^{j},R_{6}^{j}\right\}\epsilon R^{6\times N}$ of point *j* can be calculated as follows:

(9) $$R_{i,t}^{j}=\sum_{n=0}^{N-1}S_{i,j+n}\times S_{i,j+n+t}$$

As shown in Figure 5a, calculating the autocorrelation of each one-dimensional data results in six autocorrelation sequences, which are designated as channels 1–6. The autocorrelation has a local maximum (peak) in each cycle. The peak closest to the origin, which is circled by red dots in the figure, is sought for each autocorrelation sequence. If the peak is greater than the threshold (set to half of the inner product above), then all points before the peak are taken as the first single-cycle signal. Since there are six autocorrelation sequences, there are six peaks. From these six, the real peak is selected by voting. Voting means that the peak is determined to be a valid peak only when the distance between the endpoints of two or more cycles is within 10 sampling points. For example, in the left part of Figure 5a, the peaks of channel 1 and channel 2 are close and pass the blue line. Therefore, they are valid. The peak of channel 6, which is far from the other peaks, is invalid. Finally, the average of the interval from these valid peaks to the origin is taken as the interval of the first single-cycle signal, which is circled by a red ellipse in Figure 5a.

**Extracting the single-cycle signal using a classifier**

**Figure 6:**
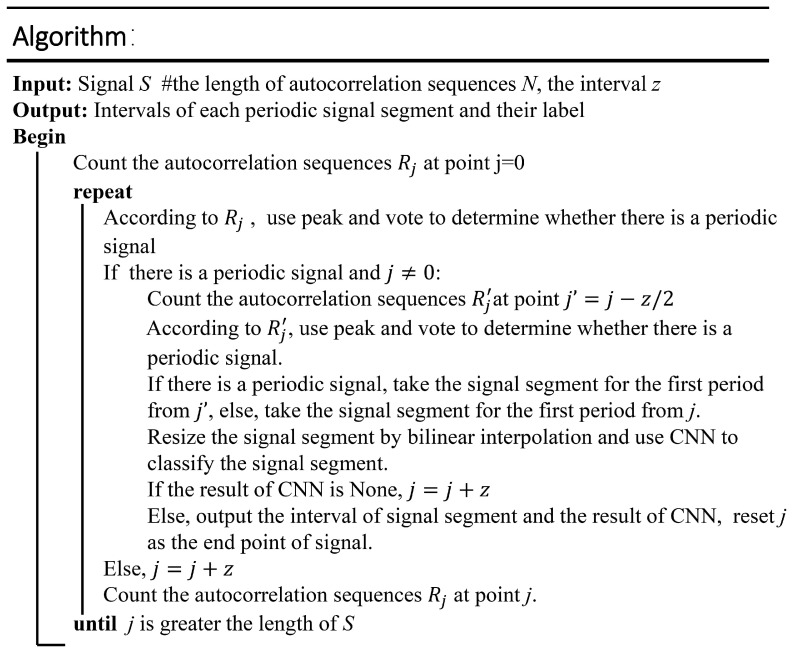
Algorithm of periodic matching based on classification.

To ensure correct periodic matching and segmentation, we combined the classification method to apply some adjustments to the periodic matching results. In general, when no periodic signal is detected, periodic matching is executed every other time gap *z* (generally set to the minimum period length of the target action). When a periodic signal is detected, it is shifted forward by z/2 lengths, and periodic matching is repeated to ensure that the obtained periodic signal spans from the beginning of the motion state to its end. As illustrated in Figure 5, if it is classified as a specific target motion state, then the next period matching starts from the end of the current period, which is framed by a red dotted box. Otherwise, it starts from the position that is z points after the start of the current period, which is framed by a yellow dotted box. The specific algorithm is shown in Figure 6.

#### 3.2.2. Classification with a Convolutional Neural Network

**Figure 7:**
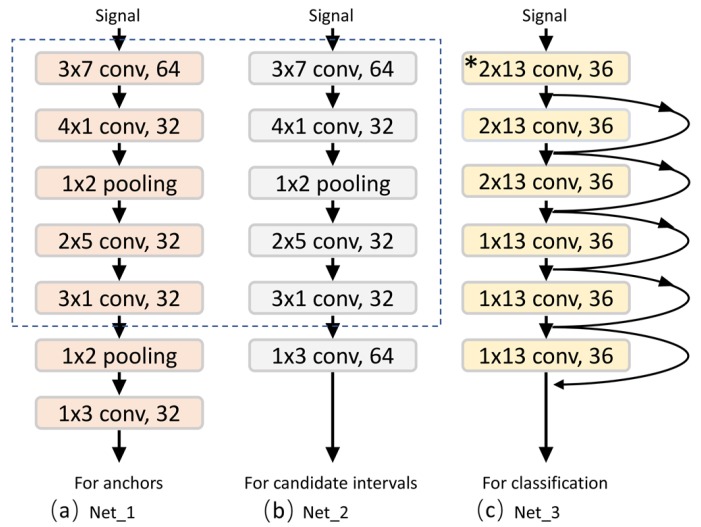
The architectures and configurations of the three networks used in this study. (**a**) Net_1 is used for interval generation in Section 3.1.1. (**b**) Net_2 is used for interval-based activity recognition in Section 3.1.2. (**c**) Net_3 is used for classification in Section 3.2.2. * indicates that the stride of the layer is 2; otherwise, it is 1. The curve with the arrow represents a “shortcut connection”.

Our convolutional classification network (Net_3 in Figure 7) is mainly composed of convolution layers, the fully connected layer, and the softmax layer. All the detected single-cycle signals are used as the input of our CNN. In addition to the four swim stroke classifications, the category None is included in the final classification result (to summarize the signals that are not suitable for the target stroke). Since we our network uses a fully connected layer, inputs to the network must be the same size. Bilinear interpolation is used to convert the input to a fixed length. The flowchart of our recognition algorithm is shown in Figure 5b. From the recognition results, we obtain the cycle counts of different motion states.

For Net_3, the cross-entropy loss is used, as in $L_{2}$.

(10) $$L_{3}=-\frac{1}{N}\sum_{i=1}^{N}\sum_{k=1}^{c+1}y_{i,k}\log p_{i,k}$$

*N* represents the number of samples, and $y_{i,c}$ is the label of the *i*th sample of the *c*th class. When the real label is the *c*th class, $y_{i,c}=1$. Otherwise, it equals 0. $p_{i,k}$ is the probability of the *i*th sample of the *c*th class, which is predicted by the CNN.

## 4. Experiments and Discussion

### 4.1. Network Setting

In this work, we used three convolutional neural networks for the detection and recognition of two types of activities. The parameters of the convolutional layers of each network are visualized in Figure 7. The two networks on the left (Net_1 and Net_2 in Figure 7a and Figure 7b) are used for the detection and recognition of the first type of activity, where the parts of the dotted line have the same structure; time is saved since they share convolutional layer parameters. The rightmost network (Net_3 in Figure 7c) is used for the recognition of the second type of activity.

In particular, since our input data size is 6*n, a horizontal rectangular convolution kernel is used to make the convolutional layer ignore the data size limit and increase its sensing field on the time axis. Note that the pooling operations in Net_1 and Net_2 are only carried out in the horizontal direction, not in the vertical direction. Because the pooling operation has a feature screening function, we opted to construct a more effective mathematical representation and capture more complex correlations in the vertical direction. We added ResNet’s [34] “shortcut connection” to the third network to make it easier to train and more accurate.

The above three convolutional neural networks were all implemented in TensorFlow. All the experiments were conducted using a regular workstation (CPU: Intel(R) Core™ i7-7700K CPU @ 4.20 GHz × 8; RAM: 16 GB).

The two activity recognition algorithms proposed in this paper are based on different detection methods, so the evaluation criteria are different. We discuss them separately in the following sections.

### 4.2. Experiments for NP_CMS Activity Detection and Recognition

The detection and recognition of NP_CMS activity is similar to the object detection task in computer vision. We used the mean average precision (mAP), which is commonly used in object detection, to evaluate our recognition method.

In the following, a “match” means that the IOU between a detection interval and a real interval with the same label is greater than 0.5.

- TP—True Positive, the number of detection results that match the real results;
- FP—False Positive, the number of detection results that do not match the real results;
- FN—False Negative, the number of real results that do not match the detection results;
- Precision—The proportion of correct detection results to all detection results, $P=\frac{TP}{TP+FP}$;
- Recall—The proportion of correct detection results to all real results, $R=\frac{TP}{TP+FN}$;
- mAP—The average accuracy under different recall rates, $mAP=\int_{0}^{1}P\left(R\right)dx$.

We divided the badminton dataset into four subsets, each of which contained data for two men and one woman. One subset was taken as the test set, and the other three were used as the training set. For the first step in network training, Net_1 was trained until the loss was stable. The second step was to train Net_1 and Net_2 together. In particular, for Net_2, our input is not only the candidate intervals generated by Net_1 but also the ground truth (or its mild deviation). This ensures that when Net_1 fails to generate high-quality candidate intervals temporarily, Net_2 guarantees that there are still high-quality candidate intervals as input.

#### 4.2.1. Evaluation of the Quality of Interval Generation

We evaluated the performance of interval generation by verifying the quality of the generated candidate intervals, which were taken as the output. The candidate intervals were then sorted with their fg parameters. Finally, the first five outputs (if the number of candidate intervals was greater than 5) were taken as the final output. In particular, we calculated the recall rate of the proposed method rather than mAP because the goal of Net_1 is to generate as many candidate intervals as possible that match the actual interval. The results are shown in Table 1. The recall rate reached 95.68%, indicating that our method generated high-quality candidate intervals.

#### 4.2.2. Comparison with Faster R-CNN

Target detection and recognition is mainly used in computer vision. Our aim is to apply it to time series. Therefore, mature algorithms for comparison with HADR are lacking. In this study, we compared our interval-based HADR with Faster R-CNN.

**Figure 8:**
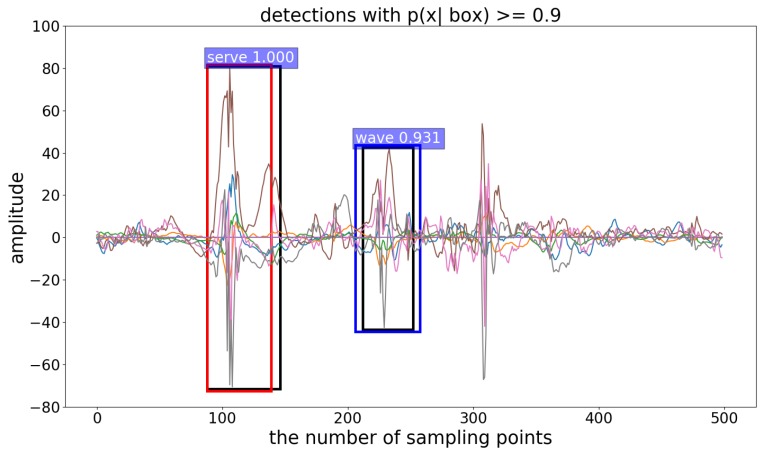
Visualization of the results. The black boxes are the real interval, the red box is the predicted interval of “serving”, and the blue box is the predicted interval of “swinging”. Their confidence levels are marked in the upper left corner.

Faster R-CNN is an algorithm for target detection and recognition in computer vision. In order to use it with time series, we converted the temporal sequence into multiple graphs with a fixed size. Compared with Faster R-CNN, our interval-based HADR achieved better results, as shown in Table 2. The average mAP of our interval-based method was 87.26%, and that of Faster R-CNN was 69.93%. These results indicate that the candidate interval captured the temporal motion characteristics better than the candidate region. The detection and recognition results are illustrated in Figure 8. Our interval-based method performed well in the detection and recognition of the target motion state.

#### 4.2.3. Comparison with Traditional Algorithms

In addition to comparing our method with Faster R-CNN, we also compared our method with two traditional sliding window methods.

**Figure 9:**
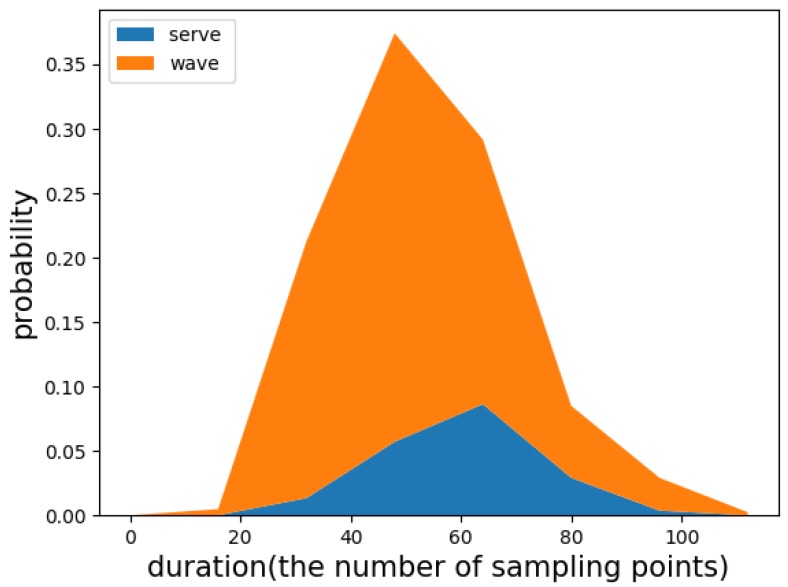
The duration distribution of the target motion states in badminton.

In the traditional sliding window method, the appropriate window length must be selected. The statistics of the durations of the two target motion states are presented in Figure 9. The durations vary widely. To improve the match of the sliding window with the duration, we used two approaches to setting the window length. The first was to use a set of sliding windows (ASSW) with different lengths and fixed overlap, and the second was to use a fixed-length sliding window (FLSW).

For ASSW, we specially trained a classifier for each length of the sliding window to identify whether the sliding window matched “serving”, “swinging”, or neither. During the testing, sliding windows of different lengths were used separately. According to the results of different classifiers, windows that matched the target motion states were selected and used as the detection results. The detection results were further processed by NMS to obtain the final detection results.

For FLSW, we changed the matching rule. Matching means that the entire real interval is included in the sliding window. In this case, we only needed to set a long sliding window to match all target motion states.

Sliding window setting: According to the matching rules and Figure 9, we set the lengths of the set of sliding windows to [30, 40, 50] sampling points and the overlap to 0.4. We set the fixed length of the sliding windows to 60 points and the overlap to 0.4. Their matching effect was verified.

Feature extraction (FE): For each one-dimensional data, we extracted its time-domain statistical features (mean value, energy, variance, interquartile range) and time–frequency statistical features (the sum of first-order detail coefficients and second-order detail coefficients of the wavelet transform). For different dimensional data, we calculated the correlation coefficient between two adjacent dimensional data.

Classifier: We adopted an SVM with a Gaussian kernel as a classifier. For each classifier, its own penalty parameters and the variance of the kernel function were determined by the grid search method.

The matching effects of the two traditional sliding window methods and our interval-based HADR are shown in Table 3. Our interval-based HADR achieved the highest recall rates. The ASSW performed much better than the FLSW in terms of recall rate. The ASSW uses sliding windows of different lengths, so it can fit the target motion states well. The average mAP values of these two sliding window methods were 23.8% and 23.7%, respectively. These values are far lower than those of our method, indicating that the sliding window methods incorrectly classified many non-matching windows as matching windows, in addition to detecting many correct windows. Therefore, it is necessary to generate the candidate intervals for the classifier.

We considered the window in the sliding window method to be an anchor and summarized the density and scale of the anchors used in the three methods. The results are shown in Table 4. Our method had more dense and multi-scaled preset anchors, explaining its high recall rates.

We also calculated the time consumed by the three methods to process a 10-second signal. All the methods were evaluated on the same hardware platform (CPU: Intel(R) Core™ i7-7700K CPU @ 4.20 GHz × 8; RAM: 16 GB). The time consumption is shown in Table 5. Our method required 20 ms to detect and recognize the motion state of a 10 s signal; this result is faster than ASSW but slower than FLSW. The interval-based method scores and fine-tunes the anchors by jointly training two CNN networks, so the processing time is longer compared with the fixed-length sliding window method. However, compared with ASSW, our method produced denser and multi-scaled preset anchors and consumed less time.

### 4.3. Experiments for WP_CMS Activity Monitoring

For the detection and recognition of the WP_CMS activity, we used the accuracy rate as the evaluation criterion. Although we lacked annotations for the duration of each motion state, we had annotations for the number of each motion state. The accuracy rate represents the ratio of the number of correctly classified samples to the total number of samples.

**Figure 10:**
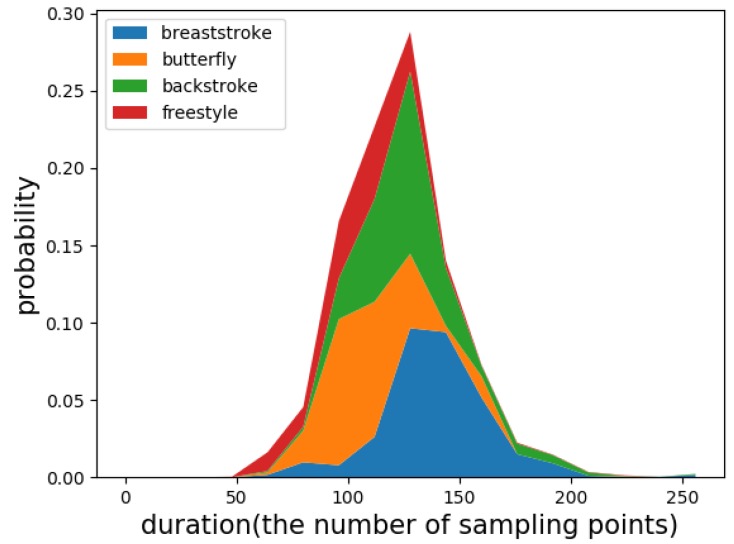
The duration distribution of four strokes.

Before the experiment, we analyzed the the duration distribution of the four swimming strokes, as shown in Figure 10. The results indicated that the duration distribution was not sufficiently concentrated. Therefore, we could not identify and count each motion state by the sliding window method.

The data of 12 participants were used as the training set, and the data of the remaining 4 were used as the test set. To verify the effectiveness of our detection and recognition algorithm, we used five classifiers to facilitate periodic matching. The features mentioned in Section 4.2.3 were used for the four classifiers other than the CNN. Before feature extraction, our periodic matching method was used to segment the signals. The results are shown in Table 6. All methods achieved the best performance, which suggests that the periodic matching method is suitable for most classifiers. The convolution operation constructs a more effective mathematical representation for the input signal. Even without feature extraction, the CNN also performed well.

We also compared the performance of the methods with and without periodic matching. For the methods that did not use periodic matching, we used a fixed-length window to segment the sensor signals. According to the distribution of the stroke duration in Figure 10, we set the window length to 2.5 s and the overlap to 0.5. As shown in Table 6, the periodic matching method improved the performance of the classifier because it provided the classifier with a complete motion cycle signal as input.

We calculated the time spent by each classifier to detect and recognize a 10 s signal, as shown in Table 7. Although the accuracy of the CNN was close to that of the other classifiers, it consumed 37 ms, which far exceeds the time consumption of the other classifiers. For the CNN design, the next step is to consider model clipping and compression. The order of time consumption (from least to most) is SVM, naive Bayes, random forest, and KNN. These methods required 11, 12, 13 and 15 ms, respectively.

**Figure 11:**
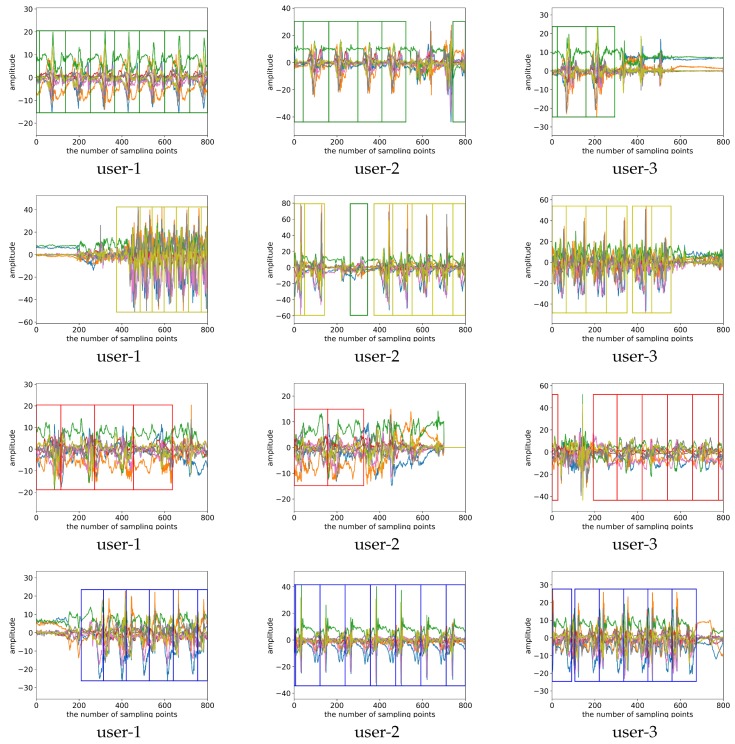
Visualization of the detection and recognition results of our proposed algorithm. The green box indicates the detected breaststroke interval, the yellow box indicates the detected butterfly interval, the red box indicates the detected backstroke interval, and the blue box indicates the detected freestyle interval.

Since our swimming dataset was not marked during the collection process, we demonstrate the performance of our algorithm by visualizing the recognition results in Figure 11.

## 5. Conclusions

In this paper, we focus on the detection and recognition of two types of activities, namely, the non-periodic activity with complex motion states (NP_CMS) and the weakly periodic activity with complex motion states (WP_CMS). In contrast to many existing HAR methods, we consider the sport-related activity monitoring problem to be a human activity detection and recognition (HADR) task that first detects meaningful motion states and then identifies them. For NP_CMS, we propose a candidate interval generation (detection) and interval-based activity recognition method. On our own dataset, the average recall of candidate interval generation was 95.68%, which means that it provided accurate candidates to the subsequent classifier. The mAP of our method was 87.3%, which indicates that the proposed interval-based activity recognition method was also very powerful. This result is better than the results of two sliding methods (ASSW and FLSW).

Classification-based periodic matching is proposed for detecting and recognizing WP_CMS. The periodic matching method can be used with many classifiers; we experimented with the CNN, SVM, naive Bayes, random forest, and KNN classifiers, and we achieved good results. With periodic matching, the duration of each motion state was accurately segmented and the input to the classifier changed from a signal with a fixed window length to a signal with only one complete period. The experimental results verify that the periodic matching improved the performance of the classifiers.

The experimental results were obtained on two relatively small datasets with few participants. Therefore, the results may be lower on a larger dataset. Moreover, the performance of the proposed methods must be verified in real-time applications and in actual life. Thus, for our next step, we plan to deploy the proposed methods on a smartwatch to evaluate their performance in real-world environments.

## Figures and Tables

**Table 1:** The recall (%) of our candidate intervals.

	Fold-1	Fold-2	Fold-3	Fold-4	Average
Recall rate	97.78	96.28	90.94	97.73	**95.68**

**Table 2:** The mean average precision (mAP) (%) of the two methods.

Method	Fold-1	Fold-2	Fold-3	Fold-4	Average
$FasterR-CNN^{\left[16\right]}$	67.72	67.13	76.47	68.38	**69.93**
Our interval-based HADR	87.87	86.63	85.48	89.07	**87.26**

**Table 3:** Comparison with sliding window methods. Feature extraction and a support vector machine (SVM) classifier were used for a set of sliding windows (ASSW) and a fixed-length sliding window (FLSW).

Method	Recall of Serving					Recall of Swinging					mAP				
	F-1	F-2	F-3	F-4	Ave	F-1	F-2	F-3	F-4	Ave	F-1	F-2	F-3	F-4	Ave
ASSW	36.6	83.3	90.0	88.0	**74.5**	83.1	92.0	94.6	92.4	**90.5**	10.3	25.3	30.2	29.3	**23.8**
FLSW	36.6	72.0	50.0	72.0	**57.6**	67.6	62.1	75.7	72.3	**69.5**	14.3	25.7	23.7	30.9	**23.7**
Our interval-based HADR	**100**	**96.0**	**90.0**	**100**	**96.5**	**95.6**	**96.6**	**91.9**	**95.5**	**94.9**	**87.9**	**86.6**	**85.5**	**89.1**	**87.3**

**Table 4:** The density and scale of anchors (unit is a sampling point).

Method	Density (Distance between Adjacent Anchors)	Scale
ASSW	12 or 16 or 20	30, 40, 50
FLSW	24	60
Our interval-based HADR	4	16, 24, 32, 40, 48, 56, 64, 72, 80, 96

**Table 5:** Comparison of time consumption.

Method	Time (ms)
ASSW	48
FLSW	11
Our interval-based HADR	20

**Table 6:** Accuracy comparison of different classifiers with and without periodic matching.

Classifier	Accuracy with Periodic Matching (%)	Accuracy without Periodic Matching (%)
CNN	97.88	93.99
SVM	97.96	94.17
Naive Bayes	96.06	91.80
Random Forest	96.97	92.80
KNN	96.67	93.89

**Table 7:** Time consumption of different classifiers with periodic matching.

Classifier	Time (ms)
CNN	37
SVM	11
Naive Bayes	12
Random Forest	13
KNN	15

## Abbreviations

The following abbreviations are used in this manuscript:

HAR	Human activity recognition
HADR	Human activity detection and recognition
CNN	Convolutional neural network
NP_CMS	Non-periodic activity with complex motion states
WP_CMS	Weak periodicity and complex motion states
PAMAP2	Physical Activity Monitoring for Aging People dataset
DSA	Daily and Sports Activity dataset
IOU	Intersection-over-union
FC	Fully connected
KNN	K-nearest neighbors
NB	Naive Bayes
RF	Random forest
SVM	Support vector machine
